# Bradykinin sensitizes the cough reflex via a B_2_ receptor dependent activation of TRPV1 and TRPA1 channels through metabolites of cyclooxygenase and 12-lipoxygenase

**DOI:** 10.1186/s12931-019-1060-8

**Published:** 2019-06-06

**Authors:** Fajer Al-Shamlan, Ahmed Z. El-Hashim

**Affiliations:** Department of Pharmacology and Therapeutics, Faculty of Pharmacy, Kuwait University, P.O. BOX 24923, 13110 Safat, Kuwait

**Keywords:** Cough, Bradykinin, B_2_ receptors, TRPV1, TRPA1, Central sensitization

## Abstract

**Background:**

Inhaled bradykinin (BK) has been reported to both sensitize and induce cough but whether BK can centrally sensitize the cough reflex is not fully established. In this study, using a conscious guinea-pig model of cough, we investigated the role of BK in the central sensitization of the cough reflex and in airway obstruction.

**Methods:**

Drugs were administered, to guinea pigs, by the intracerebroventricular (i.c.v.) route. Aerosolized citric acid (0.2 M) was used to induce cough in a whole-body plethysmograph box, following i.c.v. infusion of drugs. An automated analyser recorded both cough and airway obstruction simultaneously.

**Results:**

BK, administered by the i.c.v. route, dose-dependently enhanced the citric acid-induced cough and airway obstruction. This effect was inhibited following i.c.v. pretreatment with a B_2_ receptor antagonist, TRPV1 and TRPA1 channels antagonists and cyclooxygenase (COX) and 12-lipoxygenase (12-LOX) inhibitors. Furthermore, co-administration of submaximal doses of the TRPV1 and TRPA1 antagonists or the COX and 12-LOX inhibitors resulted in a greater inhibition of both cough reflex and airway obstruction.

**Conclusions:**

Our findings show that central BK administration sensitizes cough and enhances airway obstruction via a B_2_ receptor/TRPV1 and/or TRPA1 channels which are coupled via metabolites of COX and/or 12-LOX enzymes. In addition, combined blockade of TRPV1 and TRPA1 or COX and 12-LOX resulted in a greater inhibitory effect of both cough and airway obstruction. These results indicate that central B_2_ receptors, TRPV1/TRPA1 channels and COX/12-LOX enzymes may represent potential therapeutic targets for the treatment of cough hypersensitivity.

**Graphical abstract:**

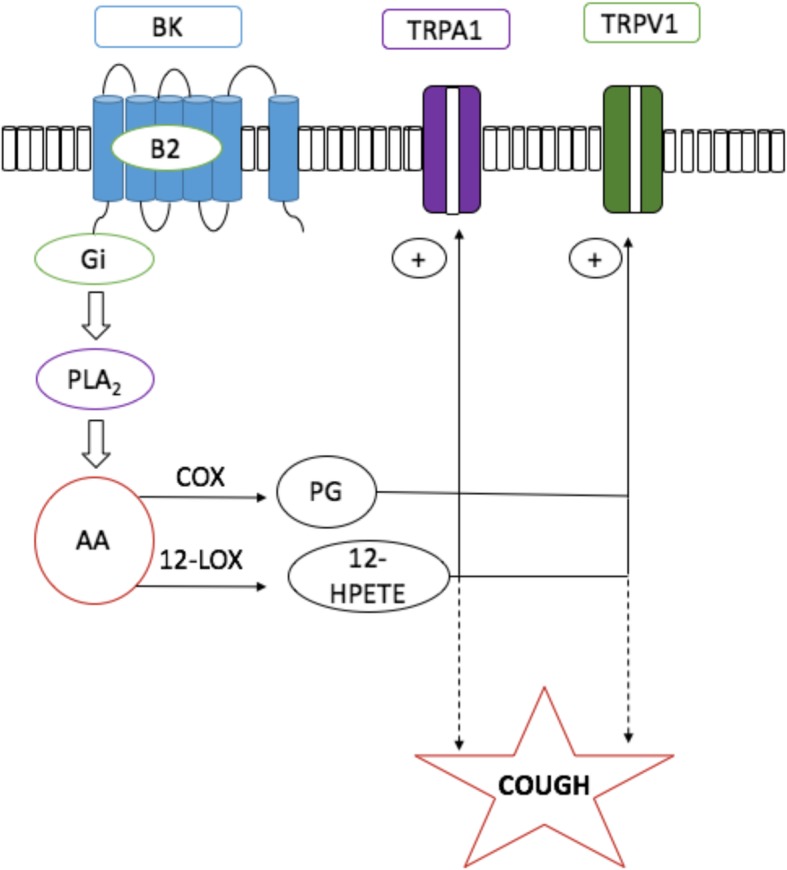

## Background

Chronic cough is a poorly understood and managed clinical problem with a high prevalence rate [21, 88]. Recently, sensitization of the cough reflex has been identified as an important mechanism in chronic cough, where cough can result from low level stimulation by chemical, mechanical, or thermal stimuli [12, 22]. The term cough hypersensitivity syndrome (CHS) has been coined to describe this phenomenon [73]. The mechanisms underlying the CHS are not yet fully understood but there is evidence to suggest that sensitization takes place at both peripheral and central levels [1, 23, 25, 30, 31]. The evidence for the involvement of peripheral sensory nerves in CHS is substantial. Numerous studies have demonstrated, using both ex vivo nerve set-ups and in vivo animal models of cough, that exposure to agents such as allergens, ozone and several inflammatory mediators result in both increased airway nerve activity  and enhanced cough [39, 53, 63]. Given that cough is predominantly vagally mediated and that the same agents which enhance afferent vagal nerve activity also sensitize the cough reflex, the role of peripheral sensitization in cough is now well established [15].

The role of the central nervous system (CNS) in cough is not well understood, mainly due to the limited access and the complexity of CNS, and possibly due to focus on the airways as the primary site for sensitization of cough. Strong evidence shows that pain, which shares many similarities with cough in terms of neuronal pathways and neurophysiology, has a strong central component [8, 14, 71]. Furthermore, drugs, both old and new ones, that effectively target pain pathways, are mainly centrally acting [85, 90]. Of relevance is that centrally acting analgesic drugs have been shown to be effective cough suppressants [4]. In addition, some mediators involved in pain pathways, such as nerve growth factor (NGF) can sensitize the cough reflex by both peripheral and central mechanisms [30, 31, 33].

Bradykinin (BK) is a well-established inflammatory mediator of both acute and chronic pain [14, 57, 82]. BK has also been reported to be involved in cough. For example, inhalation of BK or pretreatment with angiotensin converting enzyme inhibitors have been shown to induce cough and/or sensitize the cough response following challenge with tussigenic agents-an effect that can be blocked by pretreatment with a B_2_ receptor antagonist [24, 28, 30, 36, 50, 51, 67]. However, whether BK can sensitize the cough reflex via a central mode of action is not well established.

Recently, it has been reported, using an anesthetized rabbit model of cough that BK, microinjected into the nTS, enhanced the cough reflex but did not affect respiratory parameters [24]. Anesthesia however can affect the cough response. For example, several studies investigating central cough regulation, using anesthetized animals, have reported different pharmacological effects compared to conscious animals. In this regard, tracheal and laryngeal C-fiber activation by BK and/or capsaicin are known to induce cough in conscious guinea pigs but fail to induce cough in anesthetized animals suggesting that cough in the conscious and unconscious state are regulated via different mechanisms [16, 17]. In this study, using a conscious guinea pig model of cough, we investigated 1): whether central administration of BK plays a role in the sensitization of the cough reflex and/or airway obstruction in response to citric acid inhalation and 2): the mechanisms by which BK may sensitize both cough and airway obstruction in response to citric acid inhalation.

## Methods

### Animals

In house-bred Dunkin-Hartley guinea pigs (400–600 g) of either sex were maintained under temperature controlled conditions with a 12-h light/dark cycle with free access to standard chow and water ad libitum. Animals were arbitrarily assigned to control and experimental groups.

All experimental protocols were approved by the Animal Welfare and Use of Laboratory Animals Committee in the Health Sciences Center, Kuwait University and complied with the ARRIVE guidelines and were carried out in accordance with the EU Directive 2010/63/EU for animal experiments and the National Institutes of Health guide for the care and use of laboratory animals (NIH Publications No. 8023, revised 1978).

### Implantation of chronic intracerebroventricular (i.c.v.) cannulate

The implantation procedure was performed as described in detail [31]. Guinea pigs were anesthetized with a combination of 80 mg kg^-1^ ketamine hydrochloride and 6 mgkg^-1^ xylazine hydrochloride (i.m.). The depth of anesthesia was assessed by checking for the presence of the toe-pinch reflex. Supplemental anesthesia was administered in case of any reflex response. Ten min thereafter, each animal was injected with subcutaneous 0.25 mg kg^-1^ antibiotic enrofloxacin and intramuscular tramadol hydrochloride 1 mg kg^-1^; the animals were then placed in a stereotaxic apparatus (David Kopf Instruments, Tujunga, CA., U.S.A.). A midline incision in the skin above the skull (2 cm) was made with a sharp surgical blade No. 20, and the skull was cleaned with 3% hydrogen peroxide. A 20-gauge stainless steel guide cannula and its dummy cannula, HTX-20 T and HTX-25R were placed in the lowering arm of the stereotaxic apparatus. The cannula was moved in the following three dimensions relative to the bregma and corresponded to the lateral ventricles: 2.0 mm anteroposterior (AP), 1.8 mm mediolateral (ML) and 4.8 mm dorsoventral (DV), which is based on previously published work [31]. A small hole was drilled in the skull, based on the predetermined coordinates. Two additional holes were drilled for two anchor screws. Dental cement was used to fix the cannula in place. Surgical suturing was performed, using stainless steel surgical needles with 36 mm cutting edge and silk non-absorbable surgical sutures. The animals were subsequently treated with tramadol hydrochloride 1 mg kg^-1^ (i.m.) and enrofloxacin 0.25 mg kg^-1^ (s.c.), once a day for 3 consecutive days.

### Intracerebroventricular drug administration

Drugs were administered as previously described [29]. Briefly, prior to cough assessment, the dummy cannula was removed from the guide cannula. The infusion cannula was connected to the guide cannula and to a Hamilton syringe pump-model (Harvard Apparatus, USA) via a polyethylene tubing (PE-20). The drugs were infused at a rate of 30 μL hr.^-1^, with a maximum volume of 15 μL for 30 min followed by a 15-min absorption phase to prevent the backflow of the drug. The accuracy of the cannula implantation was randomly checked by the infusion of methylene blue at the end of some of the experiments. Furthermore, some experiments were carried out in dim light as some of the drugs were light sensitive.

At the end of the experiment, the guinea pigs were sacrificed by CO_2_ asphyxiation. CO_2_ flow rate was adjusted to 5 Lmin^− 1^ and continued until breathing had completely stopped. After that, cervical dislocation was performed to ensure death. The total number of guinea pigs used in the study was 171.

### Citric acid administration and cough measurement

Conscious, unrestrained, Dunkin Hartley guinea pigs were placed individually in a transparent plastic plethysmograph (Buxco, Troy, N.Y.) and exposed to nebulized 0.2 M aqueous citric acid for 10 min followed by 10 min observation period. The aerosol was produced by an aerogen nebulizer (DeVilbiss, Somerset, P.A., U.S.A.). The plethysmograph was also connected to a bias flow generator that supplied air at a rate of 3 L min^-1^ and withdraws air at a rate of 4 L min^-1^. The number of coughs was recorded over a 20-min period using the Buxco cough analyzer. This analyzer differentiates cough from a sneeze and has been reported to demonstrate > 99.0% correlation with manual cough counting [62].

### Measurement of enhanced pause (Penh)

Enhanced pause (Penh) was measured as previously described [32]. A pneumotachograph, with defined resistance in the wall of the main chamber acted as low-pass filter and allows thermal compensation. Briefly, the pressure differences between the main chamber of the body plethysmograph containing the animal, and a reference chamber (box pressure signal) was measured. The resulting box pressure signal was caused by volume and resultant pressure changes in the main chamber during the respiratory cycle of the animal. From these box pressure signals, the phases of the respiratory cycle, tidal volumes and an index of airway caliber and enhanced pause (Penh) which is an index of airway caliber can be calculated. Penh is a dimensionless value that reflects changes in the waveform of the box pressure signal from both inspiration and expiration (PIP, PEP) and combines it with the timing comparison of early and late expiration (Pause). This has been previously shown to correlate well with airway resistance and breathing pattern [29, 41, 52, 62]. Penh was recorded over the 20 min period using the analyzer of Buxco system.

### Experimental protocol and design

#### Effect of BK (i.c.v.) on citric acid-induced cough and Penh

Animals were arbitrarily divided into three groups (*n* = 5–9). Group 1 was treated with the vehicle of BK. Groups 2 and 3 were treated with 0.03 and 0.06 nmole ml^− 1^of BK, respectively. Fifteen min after infusion of BK or its vehicle, all animals were exposed to aerosolized citric acid (0.2 M) for 10 min. Cough and Penh were assessed during the citric acid challenge and for 10 min thereafter.

#### Effect of the pretreatment with the B_2_ receptor antagonist (i.c.v.) on BK-enhanced citric acid-induced cough and Penh

Animals were arbitrarily divided into 3 groups (*n* = 6–8). Group 1 was pretreated with the vehicle of the bradykinin antagonist (HOE-140). Groups 2 and 3 were pretreated with 10 and 100 nmole ml^− 1^ of HOE-140, respectively, and 15 min after the infusion of the antagonist or its vehicle, animals were treated with BK (0.06 nmole ml^− 1^). Fifteen min after infusion of BK, all animals were exposed to aerosolized citric acid (0.2 M) for 10 min. Cough and Penh were assessed during the citric acid challenge and for 10 min thereafter.

#### Effect of the pretreatment with the TRPV1 channel antagonist (i.c.v.) on BK-enhanced citric acid-induced cough and Penh

Animals were arbitrarily divided into three groups (*n* = 7–9). Group 1 was pretreated with the vehicle of the TRPV1 antagonist (JNJ-17203212). Groups 2 and 3 were pretreated with 1 and 3 μmole ml^− 1^of JNJ-17203212, respectively, and 15 min after the infusion of the antagonist or its vehicle, animals were treated with BK (0.06 nmole ml^− 1^). Fifteen min after infusion of BK, all animals were exposed to aerosolized citric acid (0.2 M) for 10 min. Cough and Penh were assessed during the citric acid challenge and for 10 min thereafter.

#### Effect of the pretreatment with the TRPA1 channel antagonist (i.c.v.) on BK-enhanced citric acid-induced cough and Penh

Animals were arbitrarily divided into three groups (*n* = 8–13). Group 1 was pretreated with the vehicle of the TRPA1 antagonist (HC-030031). Groups 2 and 3 were pretreated with 60 and 150 nmole ml^− 1^ of HC-030031, respectively, and 15 min after the infusion of the antagonist or its vehicle, animals were treated with BK (0.06 nmole ml^− 1^). Fifteen min after infusion of BK, all animals were exposed to aerosolized citric acid (0.2 M) for 10 min. Cough and Penh were assessed during the citric acid challenge and for 10 min thereafter.

#### Effect of the pretreatment with sub-maximal doses of TRPV1 and TRPA1 channels antagonists (i.c.v.) on BK-enhanced citric acid-induced cough and Penh

Animals were arbitrarily divided into 2 groups (*n* = 5). Group 1 was pretreated with the vehicle of JNJ-17203212/HC-030031 and groups 2 were pretreated (co-administration) with JNJ-17203212 (1 μmole ml^− 1^) and HC-030031 (60 nmole ml^− 1^) and 15 min after the infusion of the antagonists or their vehicle, animals were treated with BK (0.06 nmole ml^− 1^). Fifteen min after infusion of BK, all animals were exposed to aerosolized citric acid (0.2 M) for 10 min. Cough and Penh were assessed during the citric acid challenge and for 10 min thereafter.

#### Effect of the pretreatment with COX enzyme inhibitor (i.c.v.) on BK-enhanced citric acid-induced cough and Penh

Animals were arbitrarily divided into 3 groups (*n* = 5–10). Group 1 was pretreated with the vehicle of the COX inhibitor (indomethacin). Groups 2 and 3 were pretreated with 30 and 80 nmole ml^− 1^ of indomethacin, respectively, and 15 min after the infusion of the antagonist or its vehicle, animals were treated with BK (0.06 nmole ml^− 1^). Fifteen min after infusion of BK, all animals were exposed to aerosolized citric acid (0.2 M) for 10 min. Cough and Penh were assessed during the citric acid challenge and for 10 min thereafter.

#### Effect of the pretreatment with the 15-LOX-1(12/15- LOX) enzyme (i.c.v.) on BK-enhanced citric acid-induced cough and Penh

Animals were arbitrarily divided into 3 groups (*n* = 5–6). Group 1 was pretreated with the vehicle of 15-LOX-1 inhibitor (ML-351). Groups 2 and 3 were pretreated with 5 and 20 μmole ml^− 1^of ML-351, respectively, and 15 min after the infusion of the antagonist or its vehicle, animals were treated with BK (0.06 nmole ml^− 1^). Fifteen min after infusion of BK, all animals were exposed to aerosolized citric acid (0.2 M) for 10 min. Cough and Penh were assessed during the citric acid challenge and for 10 min thereafter.

#### Effect of the pretreatment with the 12- LOX enzyme (i.c.v.) on BK-enhanced citric acid-induced cough and Penh

Animals were arbitrarily divided into 3 groups (*n* = 5–6). Group 1 was pretreated with the vehicle of 12-LOX inhibitor (baicalein). Groups 2 and 3 were pretreated with 30 and 100 μmole ml^− 1^ of baicalein, respectively, and 15 min after the infusion of the antagonist or its vehicle, animals were treated with BK (0.06 nmole ml^− 1^). Fifteen min after infusion of BK, all animals were exposed to aerosolized citric acid (0.2 M) for 10 min. Cough and Penh were assessed during the citric acid challenge and for 10 min thereafter.

#### Effect of the pretreatment with sub-maximal doses of both COX and 12-LOX enzymes (i.c.v.) inhibitors on BK-enhanced citric acid-induced cough and Penh

Animals were arbitrarily divided into 2 groups (n = 5–6). Group 1 was pretreated with the vehicle of indomethacin/baicalein and groups 2 were pretreated (co-administration) with indomethacin (30 nmole ml^− 1^) and baicalein (30 μmole ml^− 1^) and 15 min after the infusion of the antagonists or their vehicles, animals were treated with BK (0.06 nmole ml^− 1^). Fifteen min after infusion of BK, all animals were exposed to aerosolized citric acid (0.2 M) for 10 min. Cough and Penh were assessed during the citric acid challenge and for 10 min thereafter.

### Preparation of buffers and drugs

Stock solution of BK and HOE-140 were initially prepared by dissolving in ACSF and subsequent dilutions were made using the same solvent. JNJ-17203212, HC-030031, indomethacin, ML-351, and baicalein were initially dissolved in DMSO, dilutions were made in ACSF to yield a final concentration of 70% DMSO. Citric acid (0.2 M) was dissolved in PBS. All drugs were freshly prepared for each experiment. Experiments with baicalein were carried out in dim light because of the light sensitivity.

### Statistical analysis

Number of coughs measured during a 20-min period were expressed as mean **±** standard error of the mean (SEM). Differences in Penh/time curve between groups was determined as the mean area under the curve (AUC) for the Penh values for last 15 min for each animal. Data analysis was performed blind. Data were initially tested for normality by Shapiro Wilk Test. Normally distributed data were assessed by either one-way analysis of variance ANOVA followed by Bonferroni post hoc test or by Student’s t-test. Nonparametric data were either analyzed by nonparametric Kruskal-Wallis test followed by Dunn’s multiple comparison test or by Mann-Whitney U test. IBM SPSS V. Seventeen software (Evanston, IL, U.S.A.) was used to assess both parametric and nonparametric data and at a p **<** 0.05 the differences were considered significant. All groups were time matched in each experiment, however, the group sizes were different due to several factors. These included some animals removing their cannula at different stages in the experiment, in addition to the anesthesia-induced death in some animals.

## Materials

Chemicals used were bradykinin acetate (Sigma-Aldrich, MO, U.S.A), HOE-140 (Sigma-Aldrich, MO, U.S.A), phosphate buffered saline (PBS) tablets (Sigma-Aldrich, MO, U.S.A), citric acid (Sigma-Aldrich, MO, USA), hydrogen peroxide (Sigma-Aldrich, Missouri, U.S.A,), JNJ-17203212 (Tocris, Cookson Ltd., Langford, U.K), dimethyl sulfoxide (DMSO; Sigma-Aldrich, MO, U.S.A), HC-030031 (Sigma-Aldrich, MO, U.S.A), indomethacin (Sigma-Aldrich, MO, U.S.A), ML-351 (Sigma-Aldrich, MO, U.S.A), baicalein (Sigma-Aldrich, MO, U.S.A), tramadol (Grünenthal, Aachen, Germany), enrofloxacin (Baytril®, BayerAG, Leverkusen, Germany), sodium dihydrogen phosphate (NaH_2_PO_4_, Missouri, USA), potassium chloride (KCl; BDH Laboratory, Poole, England), D (+) - glucose anhydrous, calcium chloride (CaCl_2_; Surechem, Needham Market Suffolk, England), sodium chloride (NaCl; Merck, Darmstadt, Germany), sodium hydrogen carbonate (NaHCO_3_; Merck, Darmstadt, Germany), magnesium chloride (MgCl_2_; Fluka, BioChemika, Messerschmitt, Switzerland), ketamine hydrochloride (Hikma pharmaceuticals, Amman, Jordan), xylazine hydrochloride (Sigma-Aldrich, MO, U.S.A), anchor screws (Stoelting, IL, U.S.A), dental cement (Stoelting, IL, U.S.A), stainless steel surgical needles with 36 mm cutting edge (1/2 circle)(Mani, INC, Tochigi, Japan), silk - non-absorbable surgical sutures (Look surgical specialties corporation, Pennsylvania, USA), surgical blades (Feather Safety Razar Co, Osaka, Japan), Hamilton syringe pump-model (Harvard Apparatus, USA), dual syringe pump-model 11 Plus (Harvard Apparatus, Holliston, MA, USA), polyethylene tubing (PE-20) (Small parts, Indiana, USA), HTX-20 T and HTX-25R stainless steel hypo tubes (Small parts, Indiana, USA). Twenty gauge guides and infusion cannulae were made by cutting HTX-20 T and HTX-25R hypo tubes, respectively.

## Results

### Effect of BK treatment (i.c.v.) on citric acid-induced cough and increase in Penh

**Fig. 1:**
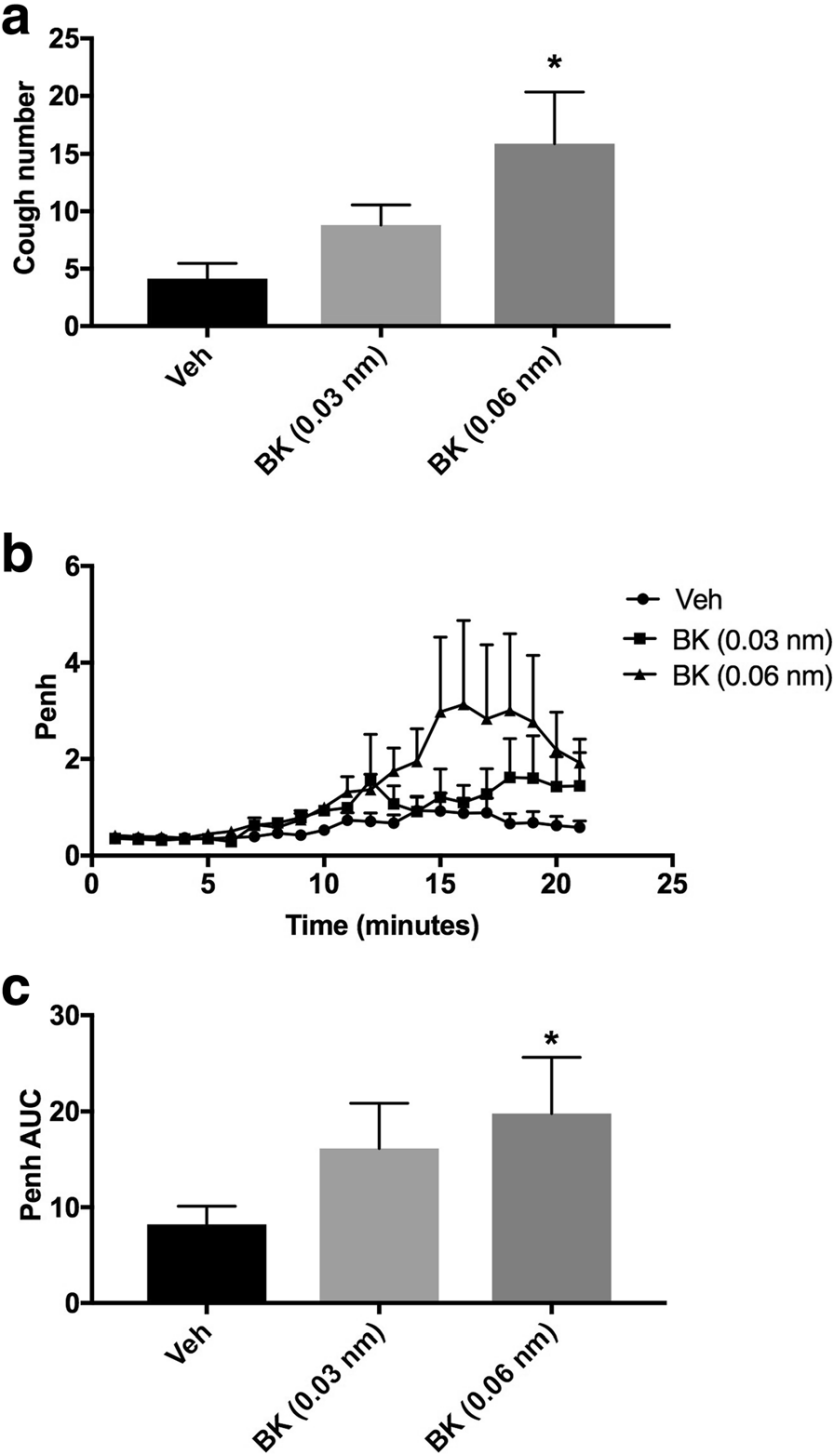
Effect of i.c.v. administered BK (0.03 and 0.06 nmole ml^− 1^; *n* = 5 and 9, respectively), versus vehicle (*n* = 8), on citric acid-induced cough (**a**) changes in Penh (**b**) and Penh AUC (**c**). Values represent means + sem. * *p* < 0.05, significant difference compared to vehicle treated animals

Administration of BK resulted in a dose-dependent increase in the citric acid-induced cough response (mean cough ± SEM: 8.8 ± 1.7 and 15.9 ± 4.5 for 0.03 and 0.06 nmole ml^− 1^ BK, respectively, compared to vehicle treated animals, 4.1 ± 1.3; Fig. 1a). BK, at 0.06 nmole ml^− 1^ significantly increased the citric acid-induced cough by more than 280% (*P* < 0.05; Fig. 1a). BK treatment also resulted in a dose-dependent enhancement in the citric acid-induced increase in Penh (mean AUC ± SEM: 16.2 ± 4.7 and 19.8 ± 5.8 for 0.03 and 0.06 nmole ml^− 1^ BK; respectively, compared to vehicle treated animals, 8.2 ± 1.9; Fig. 1b and c). BK, at 0.06 nmole ml^− 1^, significantly enhanced Penh by more than 140% (*P* < 0.05; Fig. 1b and c). Based on the significant enhancement of BK at 0.06 nmole ml^− 1^ on both cough and Penh, this dose was chosen for the rest of the experiments in this study.

### Effect of pretreatment with HOE-140 (i.c.v.) on BK-enhanced citric acid-induced cough and increased Penh

**Fig. 2:**
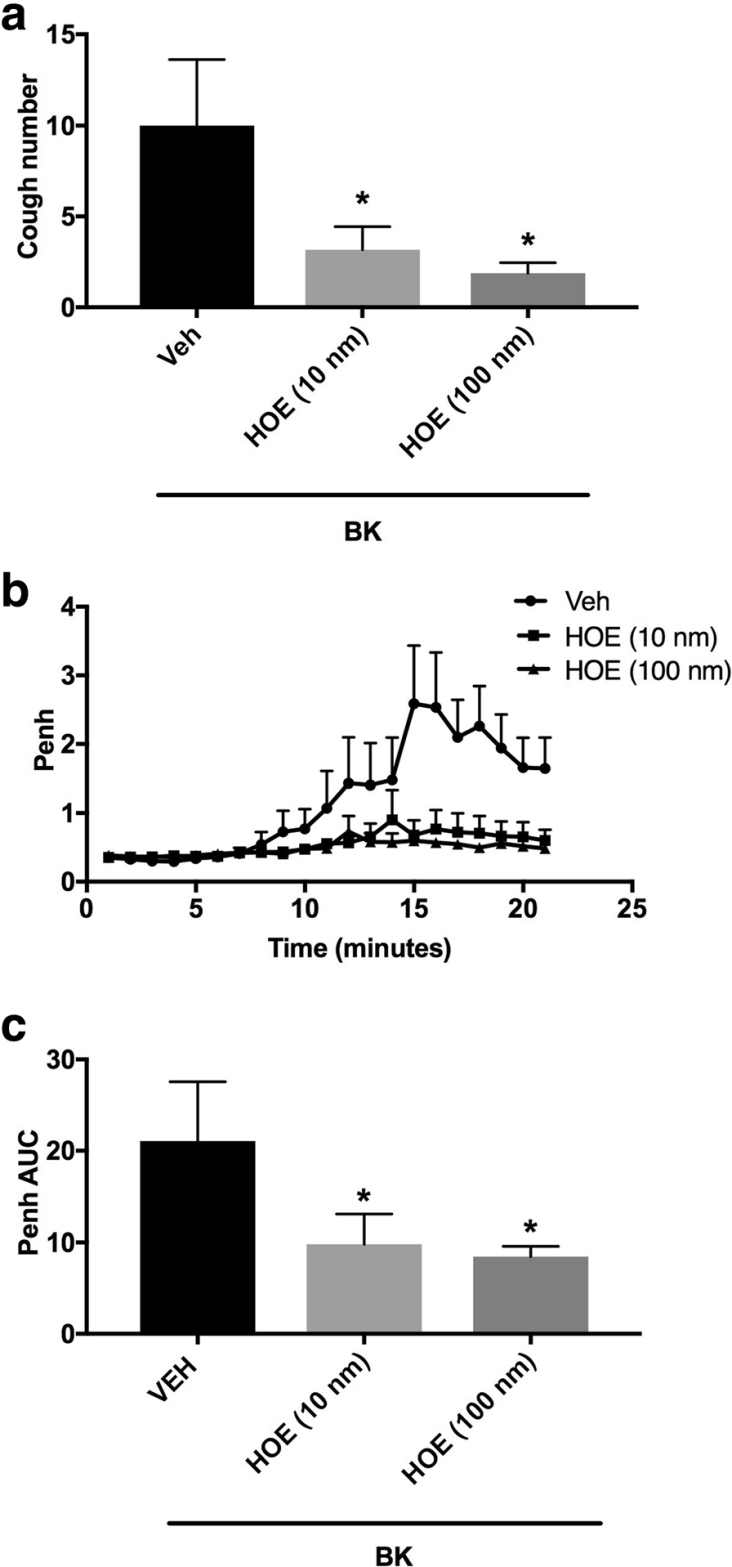
Effect of i.c.v. administered B_2_ receptor antagonist, HOE-140 10 nmole ml^− 1^ (*n* = 6) and 100 nmole ml^− 1^ (*n* = 8), versus vehicle (*n* = 7), on BK-enhanced citric acid-induced cough (**a**) changes in Penh (**b**) and Penh AUC (**c**). Values represent means + sem. * *p* < 0.05, significant difference compared to vehicle/BK treated animals

Pretreatment with the B_2_ receptor antagonist, HOE-140, resulted in a dose-dependent inhibition of the BK enhanced citric acid-induced cough (mean cough ± SEM: 3.2 ± 1.3 and 2.0 ± 0.6 for 10 and 100 nmole ml^− 1^ HOE-140; respectively, compared to vehicle pretreated animals, 10.0 ± 3.6; Fig. 2a). HOE-140, at both doses, significantly inhibited the BK-enhancement of cough response following citric acid challenge by 70 and 80% for 10 and 100 nmole ml^− 1^ HOE-140, respectively (*P* < 0.05; Fig. 2a). Pretreatment with HOE-140 also resulted in a dose-dependent decrease in the BK-enhancement of Penh following citric acid challenge (mean AUC ± SEM: 9.8 ± 3.3 and 8.4 ± 1.1 for 10 and 100 nmole ml^− 1^ HOE-140; respectively, compared to vehicle pretreated animals, 21.0 ± 6.5; Fig. 2b and c) yielding an approximate 53 and 60% decrease in the BK-enhancement of Penh following citric acid challenge for 10 and 100 nmole ml^− 1^, respectively (*P* < 0.05; Fig. 2b and c).

### Effect of pretreatment with JNJ-17203212 (i.c.v.) on BK-enhanced citric acid-induced cough and increased Penh

**Fig. 3:**
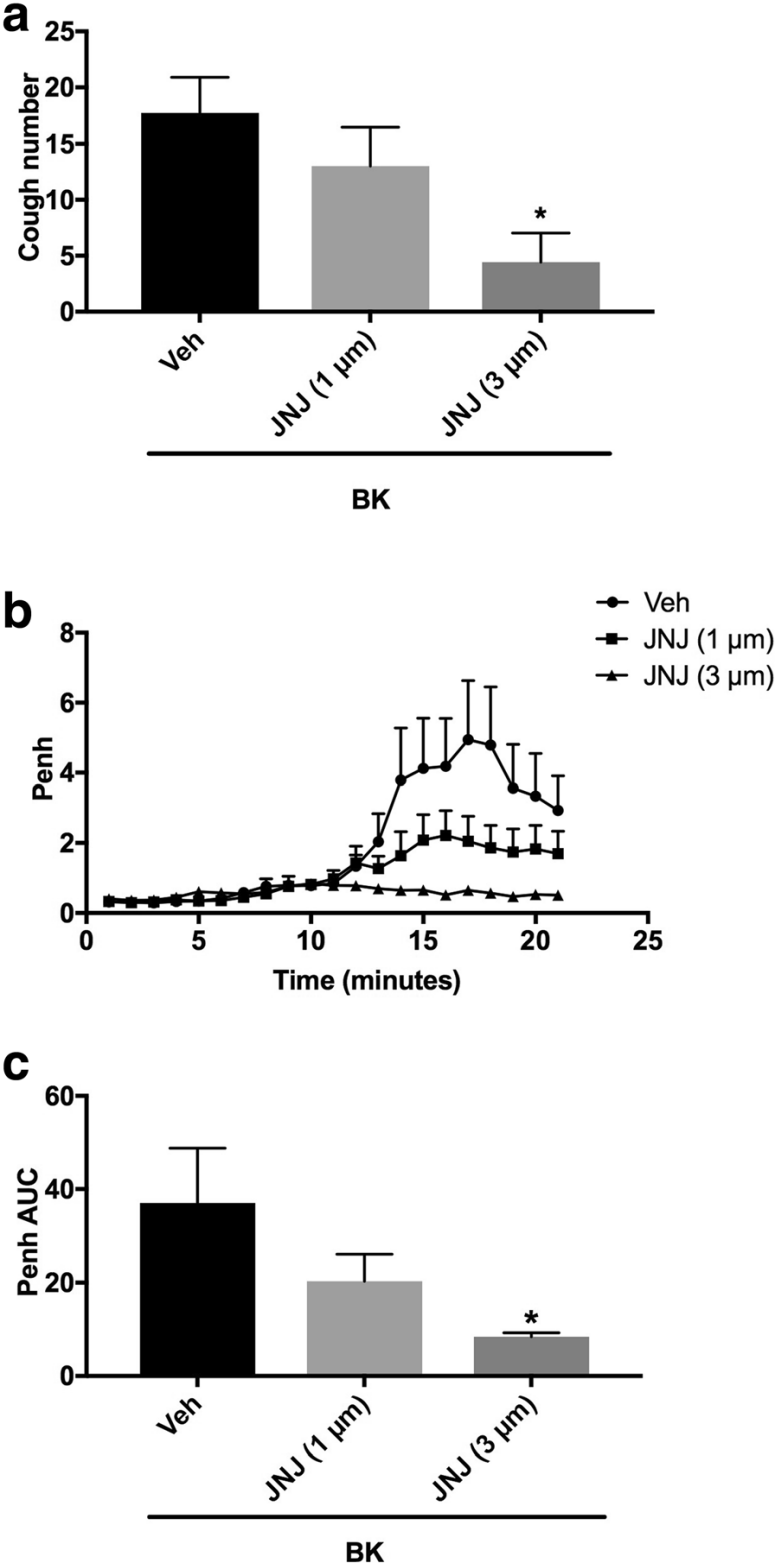
Effect of i.c.v. administered TRPV1 channel antagonist, JNJ-17203212 (JNJ) 1 μmole ml^− 1^ (*n* = 9) and 3 μmole ml^− 1^ (*n* = 7), versus vehicle (*n* = 7), on BK-enhanced citric acid-induced cough (**a**) changes in Penh (**b**) and Penh AUC (**c**). Values represent means + sem. * *p* < 0.05, significant difference compared to vehicle/BK treated animals

Pretreatment with JNJ-17203212 resulted in a dose-dependent inhibition of the BK enhanced citric acid-induced cough (mean cough ± SEM: 13.0 ± 3.5 and 4.4 ± 2.6 vs. 17.7 ± 3.2 for JNJ-17203212, 1 and 3 μmole ml^− 1^, compared to vehicle pretreated animals, respectively; Fig. 3a). Treatment with JNJ-17203212, at only 3 but not 1 μmole ml^− 1^, significantly inhibited the BK-enhancement of cough following citric acid challenge by 75% (*P* < 0.05; Fig. 3a). Pretreatment with JNJ-17203212 also resulted in a dose-dependent decrease in the BK-enhancement of Penh following citric acid challenge (mean AUC ± SEM: 20.3 ± 5.8 and 8.4 ± .0.88 vs. 37.0 ± 11.7 for 1 and 3 μmole ml^− 1^ compared to vehicle pretreated animals, respectively; Fig. 3b and c). Treatment with JNJ-17203212, at only 3 but not 1 μmole ml^− 1^, significantly inhibited the BK-enhancement of Penh following citric acid challenge by 77% (*P* < 0.05; Fig. 3b and c).

### Effect of pretreatment with HC-030031 (i.c.v.) on BK-enhanced citric acid-induced cough and increased Penh

**Fig. 4:**
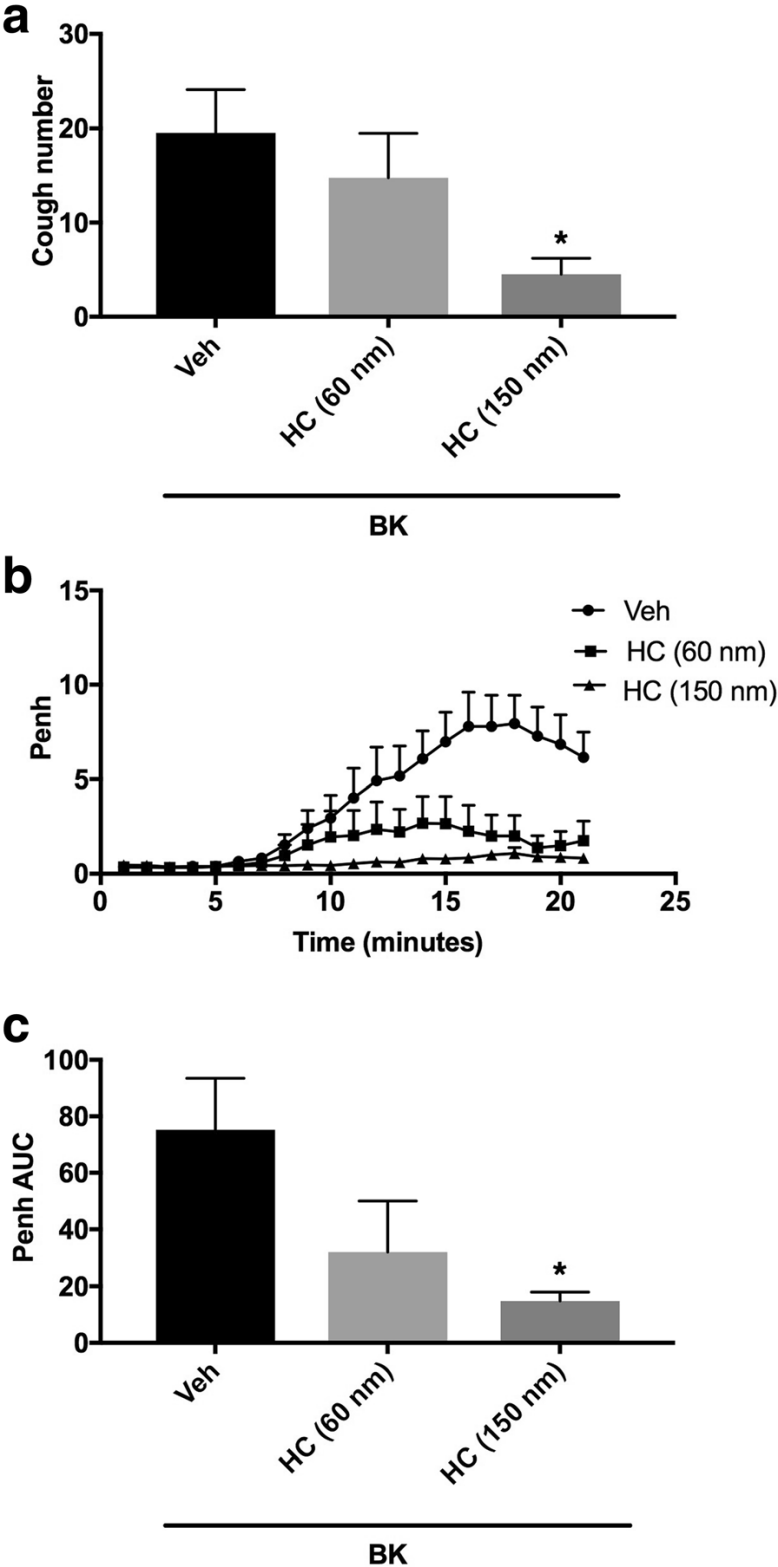
Effect of i.c.v. administered TRPA1 channel antagonist, HC-030031 (HC) 60 nmole ml^− 1^ (*n* = 8) and 150 nmole ml^− 1^ (*n* = 8)versus vehicle (*n* = 13), on BK-enhanced citric acid-induced cough (**a**) changes in Penh (**b**) and Penh AUC (**c**). Values represent means + sem. * *p* < 0.05, significant difference compared to vehicle/BK treated animals

Pretreatment with HC-030031 resulted in a dose-dependent inhibition of the BK-enhanced citric acid-induced cough (mean cough ± SEM: 14.8 ± 4.7 and 4.5 ± 1.7 vs 19.5 ± 4.5 for 60 and 150 nmole ml^− 1^ HC-030031 compared to vehicle pretreated animals, respectively; Fig. 4a). HC-030031, at 150 but not 60 nmole ml^− 1^, significantly inhibited the BK-enhancement of cough following citric acid challenge by 77% (*P* < 0.05; Fig. 4a). Pretreatment with HC-030031 also resulted in a dose-dependent decrease in the BK-enhancement of Penh following citric acid challenge (mean AUC ± SEM: 32.1 ± 17.9 and 14.76 ± 3.2 vs. 75.2 ± 18.3 for 60 and 150 nmole ml^− 1^ compared to vehicle pretreated animals, respectively; Fig. 4b and c). HC-030031, at 150 but not 60 nmole ml^− 1^, significantly inhibited the BK-enhancement of Penh following citric acid challenge by 80% (*P* < 0.05; Fig. 4b and c).

### Effect of combined pretreatment with the sub-maximal doses of JNJ-17203212 and HC-030031 on BK-enhanced citric acid-induced cough and increased Penh

**Fig. 5:**
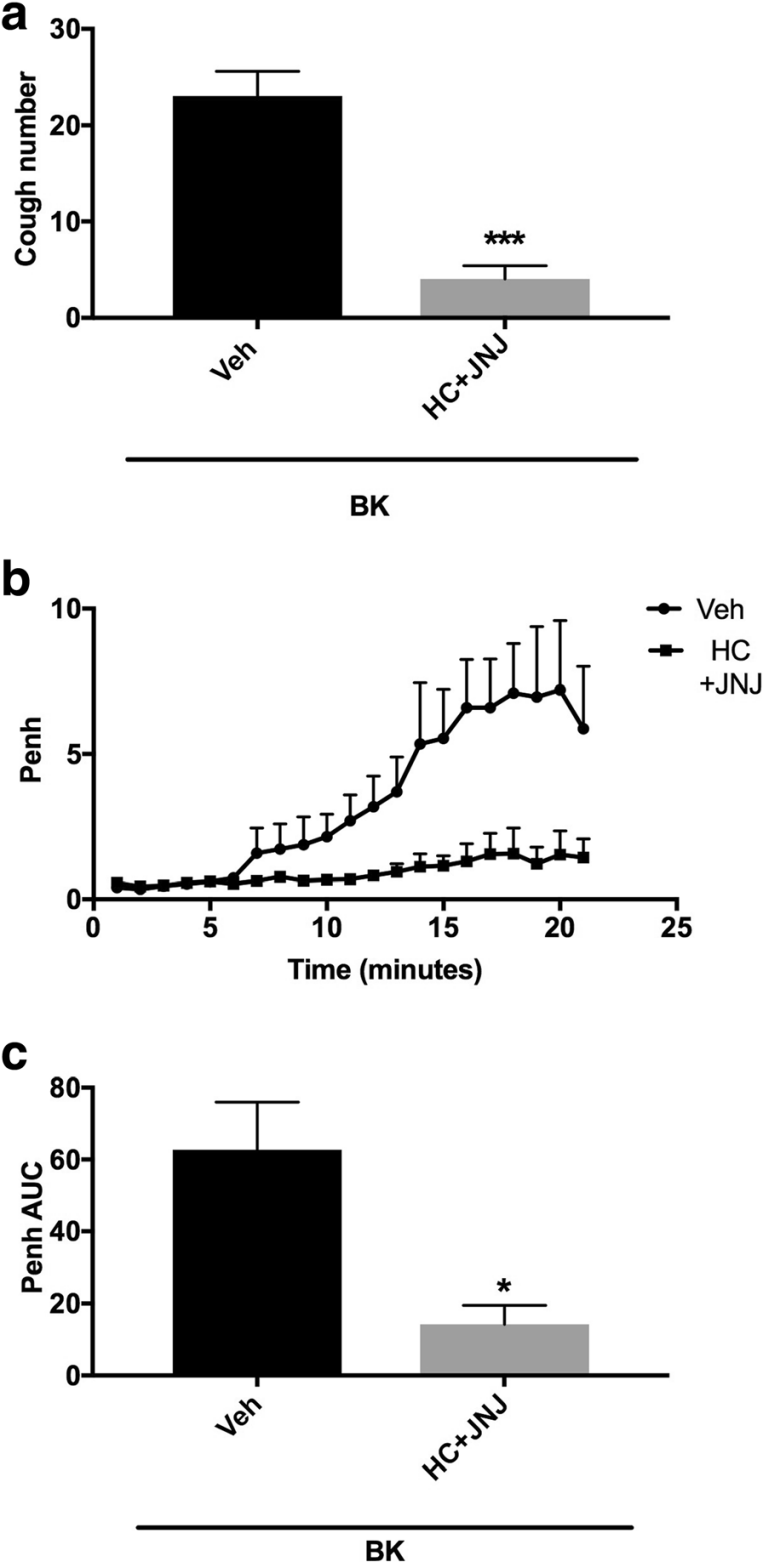
Effect of i.c.v. co-administration of TRPV1 channel antagonist, JNJ-17203212 (JNJ) 1 μmole ml^− 1^ and TRPA1 channel antagonist, HC-030031 (HC) 60 nmole ml^− 1^ (*n* = 5), versus vehicle (*n* = 5), on BK-enhanced citric acid-induced cough (**a)** changes in Penh (**b**) and Penh AUC (**c**). Values represent means + sem. * *p* < 0.05; ** *p* < 0.01; *** *p* < 0.001, significant difference compared to vehicle/BK treated animals

Pretreatment with a combination of both JNJ-17203212 (1 μmole ml^− 1^) and HC-030031 (60 nmole ml^− 1^) inhibited the BK enhancement of citric acid-induced cough by 83% (mean cough = 4.0 ± 1.4 vs 23.0 ± 2.6 compared to vehicle pretreated animals, *P* < 0.001; Fig. 5a). Similarly, pretreatment with the combined sub-maximal doses of JNJ-17203212 and HC-030031 significantly decreased the BK enhancement of the citric acid–induced increase in Penh by 77% (mean AUC ± SEM: 14.3 ± 5.2 vs. 62.7 ± 13.2 compared to vehicle pretreated animals, *P* < 0.05; Fig. 5b and c).

### Effect of pretreatment with indomethacin (i.c.v.) on BK-enhanced citric acid-induced cough and increased Penh

**Fig. 6:**
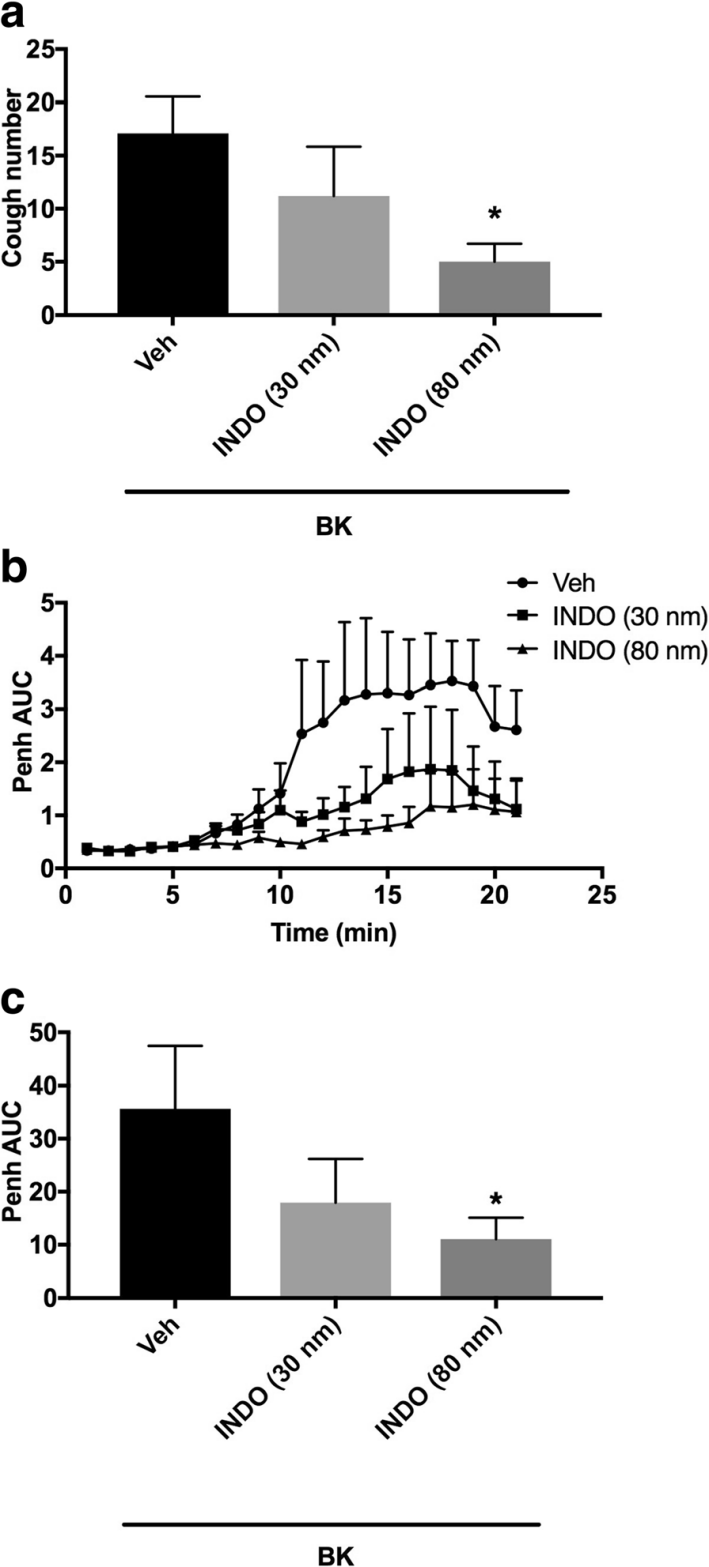
Effect of i.c.v. administered non-selective COX inhibitor, indomethacin (INDO) 30 nmole ml^− 1^ (*n* = 5) and 80 nmole ml^− 1^ (*n* = 6), versus vehicle (*n* = 10), on BK-enhanced citric acid-induced cough (**a**) changes in Penh (**b**) and Penh AUC (**c)**. Values represent means + sem. * *p* < 0.05, significant difference compared to vehicle/BK treated animals

Pretreatment with indomethacin resulted in a dose-dependent inhibition of the BK-enhanced citric acid-induced cough (mean cough ± SEM: 11.2 ± 4.6 and 5 ± 1.7 vs 17.1 ± 3.5 for 30 and 80 nmole ml^− 1^ compared to vehicle pretreated animals, respectively; Fig. 6a). Indomethacin, at 80 but not 30 nmole ml^− 1^, significantly inhibited the BK-enhancement of cough following citric acid challenge by 70% (*P* < 0.05; Fig. 6a). Indomethacin pretreatment also resulted in a dose-dependent decrease in the BK-enhancement of citric acid-induced increase in Penh (mean AUC ± SEM: 18.0 ± 8.2 and 11.1 ± 4.0 vs 35.6 ± 11.8 for 30 and 80 nmole ml^− 1^ indomethacin compared to vehicle pretreated animals, respectively; Fig. 6b and c). Indomethacin, at only 80 but not 30 nmole ml^− 1^, significantly reduced the BK-enhancement of Penh following citric acid challenge by 69% (*P* < 0.05; Fig. 6b and c).

### Effect of pretreatment with ML-351 (i.c.v.) on BK-enhanced citric acid-induced cough and increased Penh

**Fig. 7:**
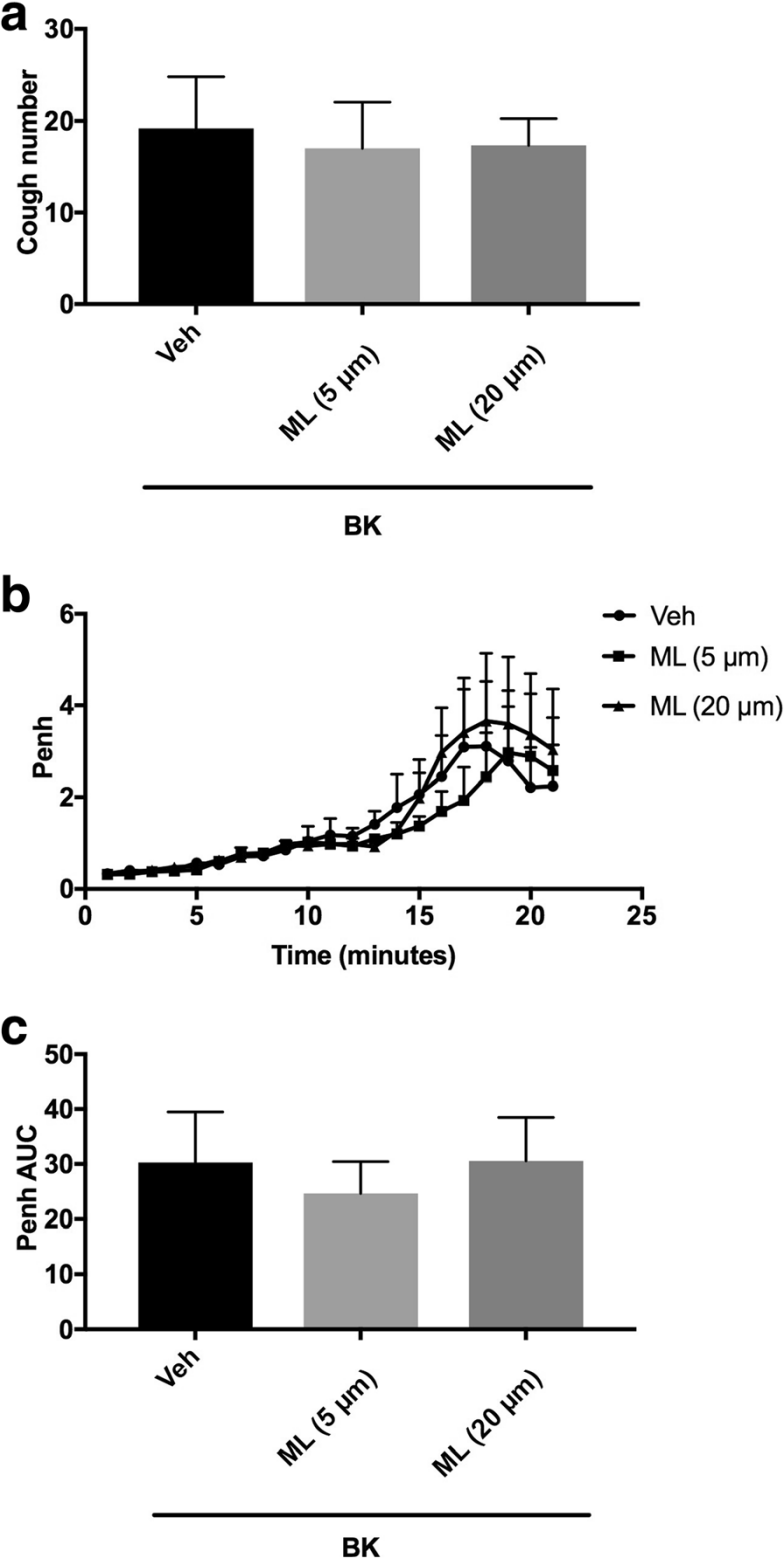
Effect of i.c.v. administered selective 15-LOX-1 inhibitor, ML-351 (ML) 5 μmole ml^− 1^ (*n* = 6) and 20 μmole ml^− 1^ (*n* = 6), versus vehicle (*n* = 5), on BK-enhanced citric acid-induced cough (**a**) changes in Penh (**b**) and Penh AUC (**c**). Values represent means + sem

Pretreatment with ML-351 did not affect the BK-enhancement of citric acid-induced cough response (mean cough ± SEM: 17.0 ± 5.0 and 17.3 ± 2.9 vs 19.2 ± 5.6 for 5 and 20 μmole ml^− 1^ ML-351 compared to vehicle pretreated animals, respectively; Fig. 7a). Similarly, ML-351 pretreatment did not affect the BK-enhancement of Penh following citric acid challenge (mean AUC ± SEM: 24.6 ± 5.8 and 30.4 ± 7.9 vs 30.3 ± 9.1 for 5 and 20 μmole ml^− 1^ compared to vehicle pretreated animals, respectively; Fig. 7b and c). Higher doses could not be tested because of the solubility limitation.

### Effect of pretreatment with baicalein (i.c.v.) on BK-enhanced citric acid-induced cough and increase in Penh

**Fig. 8:**
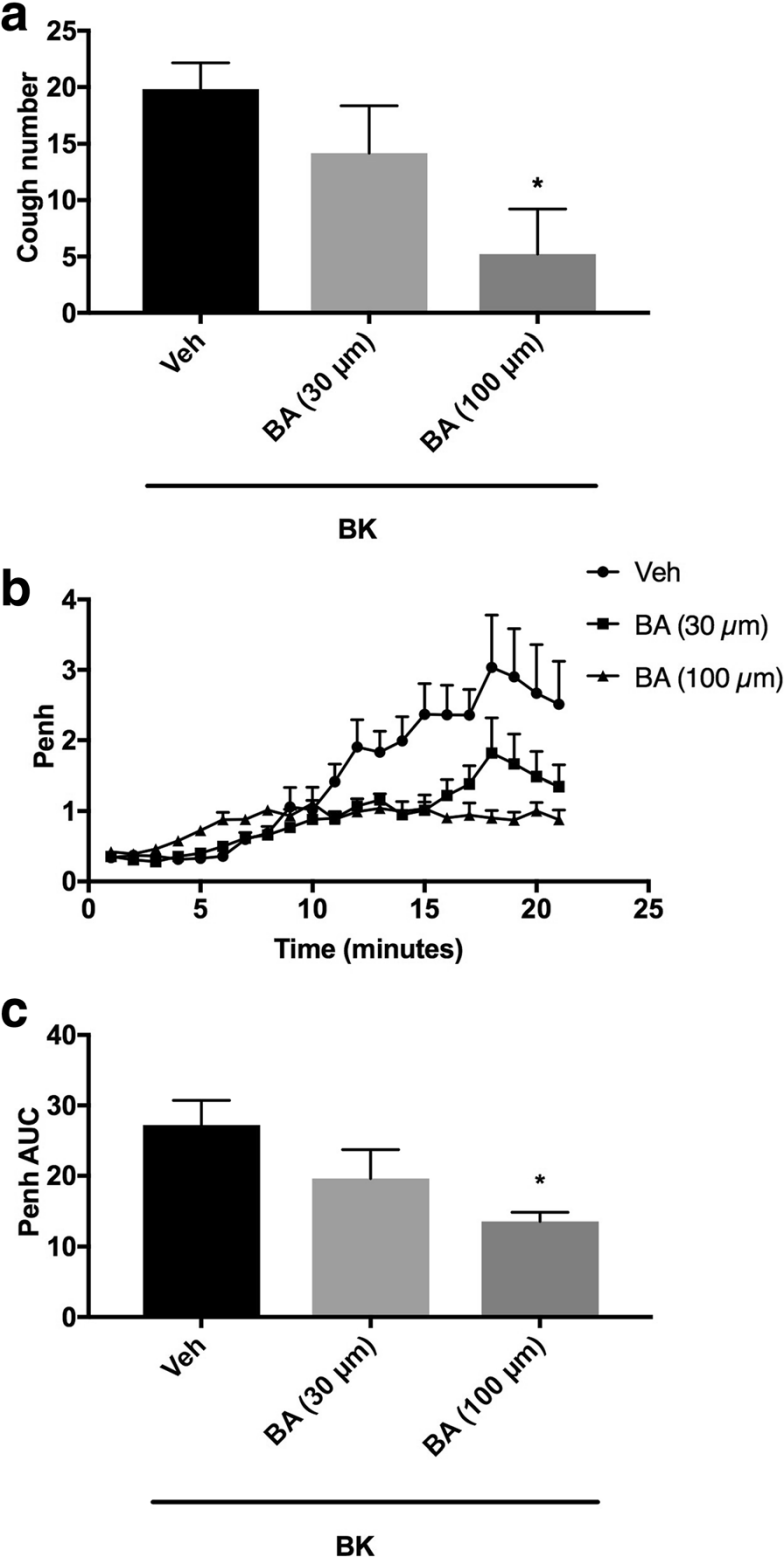
Effect of i.c.v. administered 12-LOX inhibitor, baicalein (BA) 30 μmole ml^− 1^ (*n* = 6) and 100 μmole ml^− 1^ (*n* = 5), versus vehicle (*n* = 6), on BK-enhanced citric acid-induced cough (**a**) changes in Penh (**b**) and Penh AUC (**c**). Values represent means + sem. * *p* < 0.05, significant difference compared to vehicle/BK treated animals

Pretreatment with baicalein resulted in a dose-dependent inhibition of the BK enhanced citric acid-induced cough (mean cough ± SEM: 14.2 ± 4.2 and 5.2 ± 4.0 vs 19.8 ± 2.3 for 30 and 100 μmole ml^− 1^ baicalein compared to vehicle pretreated animals, respectively; Fig. 8a). Baicalein, at 100 but not 30 μmole ml^− 1^, significantly inhibited the BK-enhancement of cough following citric acid challenge by 74% (*P* < 0.05; Fig. 8a). Pretreatment with baicalein also resulted in a dose-dependent decrease in the BK-enhancement of Penh following citric acid challenge (mean AUC ± SEM: 19.6 ± 4.1 and 13.5 ± 1.3 vs 27.2 ± 3.5 for 30 and 100 μmole ml^− 1^ compared to vehicle pretreated animals, respectively; Fig. 8b and c). Baicalein, at 100 but not 30 μmole ml^− 1^, significantly reduced the BK-enhancement of Penh following citric acid challenge by 50% (P < 0.05; Fig. 8b and c).

### Effect of combined pretreatment with the sub-maximal doses of indomethacin and baicalein (i.c.v.) on BK-enhanced citric acid-induced cough and increase in Penh

**Fig. 9:**
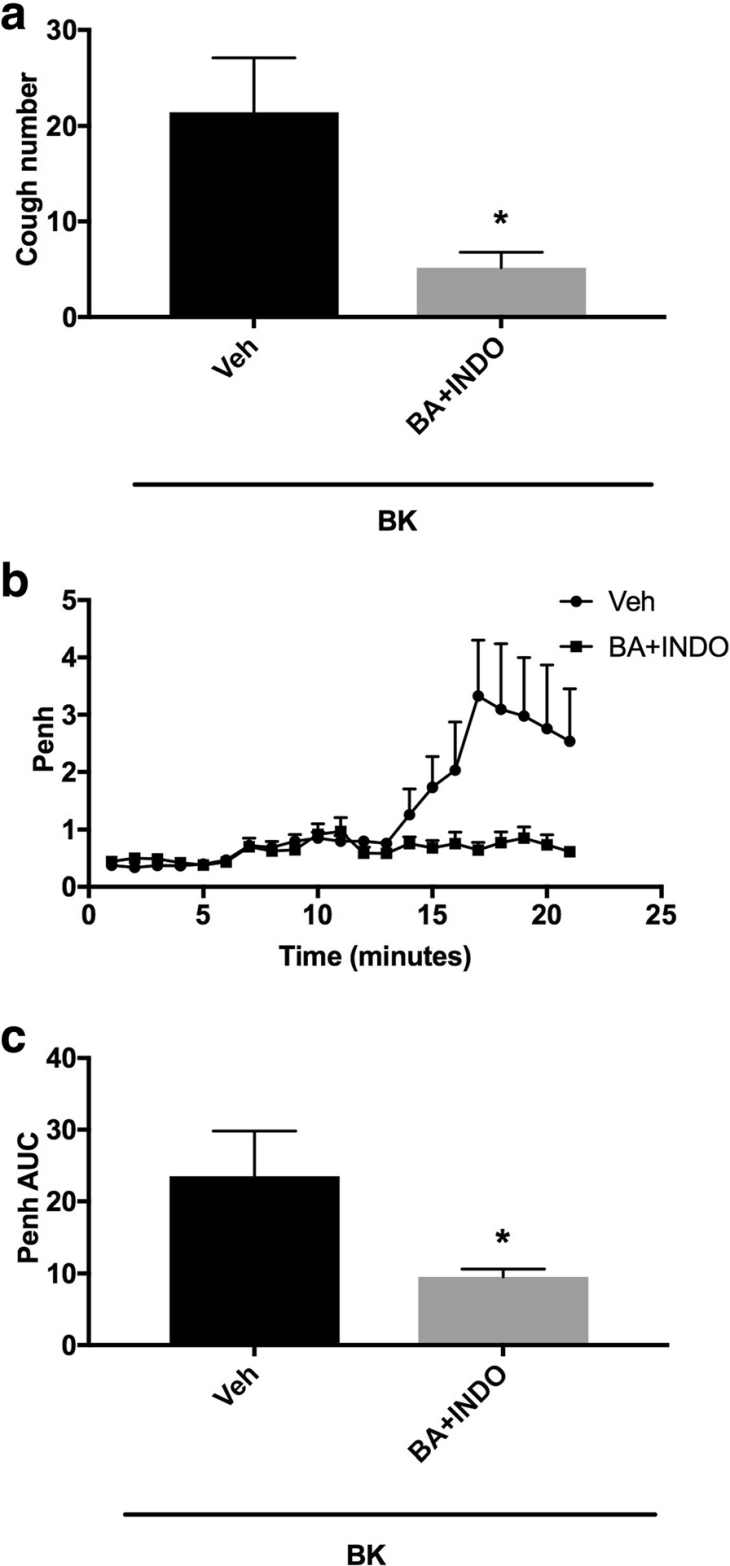
Effect of i.c.v. co-administration of non-selective COX inhibitor, indomethacin (INDO) 30 nmole ml^− 1^ and 12-LOX inhibitor, baicalein (BA) 30 μmole ml^− 1^ (*n = 6*),versus vehicle (*n* = 5), on BK-enhanced citric acid-induced cough (**a**) changes in Penh (**b**) and Penh AUC (**c**). Values represent means + sem. * *p* < 0.05 significant difference compared to vehicle/BK treated animals

Pretreatment with a combination of indomethacin (30 nmole ml^− 1^) and baicalein (30 μmole ml^− 1^) inhibited the BK-enhancement of citric acid-induced cough by 76% (mean cough = 5.2 ± 1.6 vs 21.4 ± 5.7 for the combination compared to vehicle pretreated animals, respectively, P < 0.05; Fig. 9a). Similarly, pretreatment with the combined sub-maximal doses of indomethacin and baicalein significantly decreased the BK-enhancement of citric acid-induced increase in Penh by 60% (mean AUC ± SEM: 9.5 ± 1.1 vs. 23.5 ± 6.3 for the combination compared to vehicle pretreated animals, P < 0.05; Fig. 9b and c).

## Discussion

In this study we show, using a conscious guinea-pig model of cough, that centrally administered BK sensitizes the cough reflex via B_2_ receptors. We also show that the central BK-induced sensitization occurs via activation of both TRPV1 and TRPA1 channels through metabolites of COX and 12-LOX enzymes. Furthermore, our data show that combined blockade of both TRPV1 and TRPA1 channels results in greater degree of  inhibition of both cough and airway obstruction.

BK is an important inflammatory mediator that has been shown  to be involved in cough mechanisms. Administration of BK to the airways of animals and humans results in both induction and sensitization of cough [28, 36, 39, 50, 93]. Whether BK can result in the sensitization of the cough reflex, in conscious animal models, via a central mechanism has not been addressed. Our findings show that acute exposure of BK, i.c.v., significantly increased citric acid-induced cough in a dose dependent manner, within a short period of time following  BK infusion. This adds further support to the findings showing that treatment with lisinopril up-regulates the cough reflex via a BK-dependent mechanism in an anesthetized cough model [24]_._ Our observations with BK are also similar to the cough sensitizing actions of centrally administered NGF previously reported [31]. Similarly, substance P (SP) microinjected into the nTS has been shown to up-regulate the cough reflex. These findings suggest that cough can be centrally enhanced and that central regions, particularly the nTS, represents key sites for sensitization of cough [24, 81].

Our data is also in keeping with the central effects of BK in hyperalgesia. For example, it has been shown that central administration of BK, at low doses, results in hyperalgesia 15 min later [14]. Mice lacking BK receptors also have a 70% reduction in acute acetic acid-induced nociception [20].

In addition to effects on cough, our findings also show that BK administration results in an enhancement of the citric acid-induced airway obstruction. The exact mechanism by which this may occur is not clear. However, it is possible that BK stimulates a specific set of second order neurons in the nTS which in turn activate the airway-related vagal preganglionic neuron (AVPNs). The AVPNs are the final common pathway from brain stem/CNS to the airways and transmit signals to the tracheobronchial ganglia which lie in close proximity to effector systems such as blood vessels, submucosal glands and airway smooth muscle. AVPNs are indeed central integrators of several excitatory (glutamatergic, tachykinergic) and inhibitory (GABAergic, serotonergic and noradrenergic pathways) inputs which regulate cholinergic outflow to the airways [42–49]. One possible mechanism by which BK can affect airway tone is via modulation of central SP release. Indeed, SP injected into the fourth ventricle has been shown to remarkably increase tracheal cholinergic tone [70]. Of relevance also is that i.c.v. administration of a non-selective neurokinin antagonist prevents the BK-induced potentiation of histamine-mediated increased airway cholinergic tone [70].

Our data also show that the BK effects on cough and airway obstruction are mediated mainly via B_2_ receptors since pretreatment with the selective B_2_ receptor blocker, HOE-140, significantly inhibited the BK enhanced tussive effects by approximately 80%. This is generally in agreement with studies, in both cough and pain, showing that the B_2_ receptor, a constitutively expressed receptor, mediates BK-induced tussive and nociceptive effects in normal animals, respectively [23, 24, 36, 82]. Interestingly, a study has reported a role for B_1_ receptor in enalapril-induced cough. However, in this study, the drug treatment was done over 20–30 days which may likely explain the difference between their findings and those of ours and other groups [51]. Similar to the cough effects, pretreatment with HOE-140 also significantly blocked BK-enhancement of airway obstruction indicating that this response was also mediated via the B_2_ receptor.

Our next question was whether TRP channels, specifically TRPV1 and TRPA1, were involved in the BK sensitization of cough and airway obstruction. The reason for investigating the link between BK and the TRP channels is twofold. Firstly, several studies have shown that both TRPV1 and TRPA1 channels are involved in the transducing mechanisms of cough inducing stimuli [3, 7, 11, 13, 69, 74, 75, 87]. Secondly, TRPV1 and TRPA1 channels have been shown to be critical in mediating BK-induced cough and hyperalgesia [5, 11, 18, 39]. Our data show that pretreatment with the selective and potent TRPV1 antagonist, JNJ-17203212 [10], dose dependently inhibited the BK-induced sensitization of the cough reflex. Similarly, pretreatment with HC-030031, a selective and potent TRPA1 antagonist [27], also inhibited the BK-induced sensitization of cough response in a dose dependent manner. These findings are in line with data showing a role for both TRPV1 and TRPA1 in inhaled BK-induced cough [39].

An important role for TRPV1-dependent sensitization of the cough reflex has also been previously demonstrated for NGF, in this same model, in both peripheral and central sensitization [31]. Together, these data suggest that TRPV1, and also TRPA1, channels may act as a signaling hub for stimuli that sensitize the cough reflex. This assertion is in line with the important role that both these channels play in centrally mediated hyperalgesia [2, 18, 94].

In addition to the effects on cough, our data show that blockade of both the TRPV1 and TRPA1 channels (by JNJ-17203212 and HC-030031, respectively) resulted in a significant inhibition of the BK-induced enhancement of airway obstruction by 77 and 80%, respectively. The degree of inhibition, through blockade of both these channels, was similar thus indicating that both channels are equally important in this response. The link between TRPV1 and TRPA1 activation and increased airway obstruction is not clear, However, multimodal activation of TRPV1 channels trigger increased spontaneous glutamate release within the nTS [26, 56, 78]. Furthermore, TRPA1 channels agonists have also been reported to increase CNS neuronal excitability, For example, in the spinal dorsal horn, TRPA1 agonist not only increases the frequency but also amplifies the excitatory postsynaptic currents (EPSCs) [60]. Therefore, such action, in the nTS, may result in increased activation of AVPNs and increased airway obstruction.

Based on the fact that BK-enhanced cough and airway obstruction were dependent on both TRPV1 and TRPA1 channels activation, in our next experiments we assessed whether combined treatment with sub-maximal doses of the TPRV1 and TRPA1 antagonists would achieve greater inhibitory effects [35]. Our findings show that, in contrast to the sub-maximal dose of each drug administered alone, where the degree of inhibition of cough was 26 and 24% for JNJ-17203212 and HC-030031, respectively, combined pretreatment with both antagonists significantly reduced the BK sensitization of cough by 83%. This finding is in-line with data from a previous study showing that the combined blockade of TRPV1 and TRPA1 produced a significant degree of cough inhibition compared to each individual drug [39]. This confirms that both TRPV1 and TRPA1 channels are involved in the BK-induced sensitization of the cough reflex. Similarly, our finding shows that the combination of JNJ-17203212 and HC-030031 significantly blocked BK-enhanced airway obstruction compared to each drug administered alone. Of interest, several studies have reported that both TRPV1 and TRPA1 channels are co-expressed on sensory and dorsal root ganglia (DRG) neurons [38, 59, 75, 76]. It has also been reported that TRPA1 channels can be activated via calcium inflow through TRPV1 channels suggesting that calcium influx from one channel can result in activation of the other channel [19, 54, 61, 68, 95], thus implying that these two channel are closely linked, both physically and functionally. In view of the fact that current clinical studies with TRPV1 antagonists have failed to show any significant effects [6, 58], it is tempting to speculate, based on our findings and that of others, that both TPRA1 and TPRV1 channels may need to be blocked in order to see significant inhibitory effects on cough.

Our next question was whether the coupling of B_2_ receptors to TRPV1 and TRPA1 activation was mediated by the metabolites of COX, 12-LOX and/or 15-LOX-1 enzyme. Several studies have shown that BK activation of B_2_ receptors results in prostanoid production, such as PGE_2_, by the COX enzyme [37, 92]. Furthermore, PGE_2_, which is one of the major prostanoids, seems to play an important role in BK-induced cough and pain [39, 77, 83]. Our data show that pretreatment with indomethacin blocked the BK sensitization of cough by 70% which suggests that COX metabolites are involved in the BK sensitization of cough. This finding is in agreement with studies showing that several metabolites of the COX enzymes such as PGD_2_ and PGE_2_ can induce cough in conscious animals via mainly DP_1_ and EP_3_ receptors, respectively [64–66]. Furthermore, in support of our findings, a double-blind, randomized, cross-over study showed that treatment with indomethacin, of patients who developed cough as a side effect of chronic captopril therapy, significantly inhibited their cough [34]. This suggests that metabolic products of the COX enzyme may couple the activation of B_2_ receptors to the sensitization of TRPV1 and TRPA1 channels. In support of this assertion, zaltoprofen, an NSAID, has been shown to inhibit the enhancement effects of BK on capsaicin-induced ^45^Ca^2+^ uptake in DRG neurons [86]. Also, PGE_2_ activation of isolated guinea pig sensory ganglia was partially inhibited by blockade of either TRPA1 or TRPV1, and completely inhibited in the presence of both blockers [39].

It is worthy to note that not all studies have been able to document a role for PGE_2_ in BK induced-cough. For example, meclofenamic acid pretreatment failed to attenuate the inhaled BK-evoked cough [50]. Whilst the reasons for this discrepancy are not known, a possible explanation is that some NSAIDs can actually activate TRP channels, such as TRPA1 [55]. For example, in HEK293 cells that express TRPA1 channels, extracellular application of several NSAIDs rapidly activate TRPA1 channels. Indeed, fenamates were the most potent NSAIDs in activating TRPA1 channels, an effect blocked by pretreatment with HC-030031 [55]. Interestingly, naproxen, the only NSAIDs not reported to activate TRPA1 channels, has been shown to improve viral-induced cough [1, 55, 84].

Similar to the effects on cough, pretreatment with indomethacin inhibited the BK-enhanced airway obstruction indicating that COX metabolites are involved not only in the BK-enhanced cough but airway obstruction as well. The mechanisms by which central COX metabolites result in increased airway obstruction is not known. However, PGE_2_ induced glutamate release was noted in several regions in the CNS including the nTS [9, 91]. Indeed, i.c.v. administration of PGE_2_ results in an increased c-Fos expression in several brain regions including the brainstem [72, 80]. This implies that increased PGE_2_ release can increase neuronal activity in the brain stem which may in turn affect the airway tone.

Our data show that central pretreatment with baicalein, an inhibitor of 12-LOX significantly reduced the BK-enhanced cough response by 74%. This implies that metabolites of 12-LOX activate TRP channels such as TRPV1 and TRPA1 which then result in sensitization of the cough reflex. In support of this, BK has been previously shown to activate TRPV1 receptors via a 12-HPETE-dependent mechanism. Indeed, it has also been reported that, in DRG neurons expressing B_2_ receptors, 12-lipoxygenase and TRPV1, activation of B_2_ receptors induced synthesis of 12-HPETE which was followed by TRPV1 channel opening [82]. In addition, 12-HPETE has been also shown to activate TRPA1 via hepoxilins A_3_ and B_3_ formation [40].

Our data also shows that pretreatment with baicalein significantly inhibited the BK-enhancement of airway obstruction. The mechanism by which products of central 12-LOX enhance airway obstruction is not known. However, there is evidence that, in the spinal cord, hydroxynonenal, a metabolic product of 12-LOX, activates TRPA1 via the release of SP. Therefore, it is possible that BK-induced SP release, centrally, can activate TRPA1 channels via a 12-LOX dependent pathway which may consequently enhance the airway tone [89].

Based on the fact that products of both COX and 12-LOX appear to be involved in BK sensitization of the cough reflex and airway obstruction, we asked whether combined treatment with sub-maximal doses of the COX and 12-LOX inhibitor, would achieve greater degree of inhibition than when each drug is given alone. Our data show that the combination treatment with indomethacin and baicalein reduced the BK enhanced cough response by 76% compared to 35 and 28%, respectively, with each drug administered alone. The findings confirm that metabolites of both COX and 12-LOX are involved in the coupling of B_2_ receptors to TRPV1 and TRPA1 activation. Similarly, the combined sub-maximal doses significantly blocked the BK-enhanced airway obstruction, again confirming that both COX and 12-LOX metabolites are involved in the BK sensitization of airway obstruction.

The role of 15-LOX-1 metabolites in sensitization of cough was also investigated. In our study, pretreatment with ML-351, a selective and highly potent 15-LOX-1(12/15-LOX) inhibitor [79], failed to inhibit either BK sensitization of cough or airway obstruction. This suggests that metabolites of 15-LOX-1 are not major contributors to the BK-induced central sensitization of either cough or airway obstruction. This lack of effect of ML-351 is unlikely to be dose related as the doses used in our experiment were based on doses previously shown to have clear effects [79]. Moreover, we were unable to use higher doses of ML-351 due to solubility limitation.

**Fig. 10:**
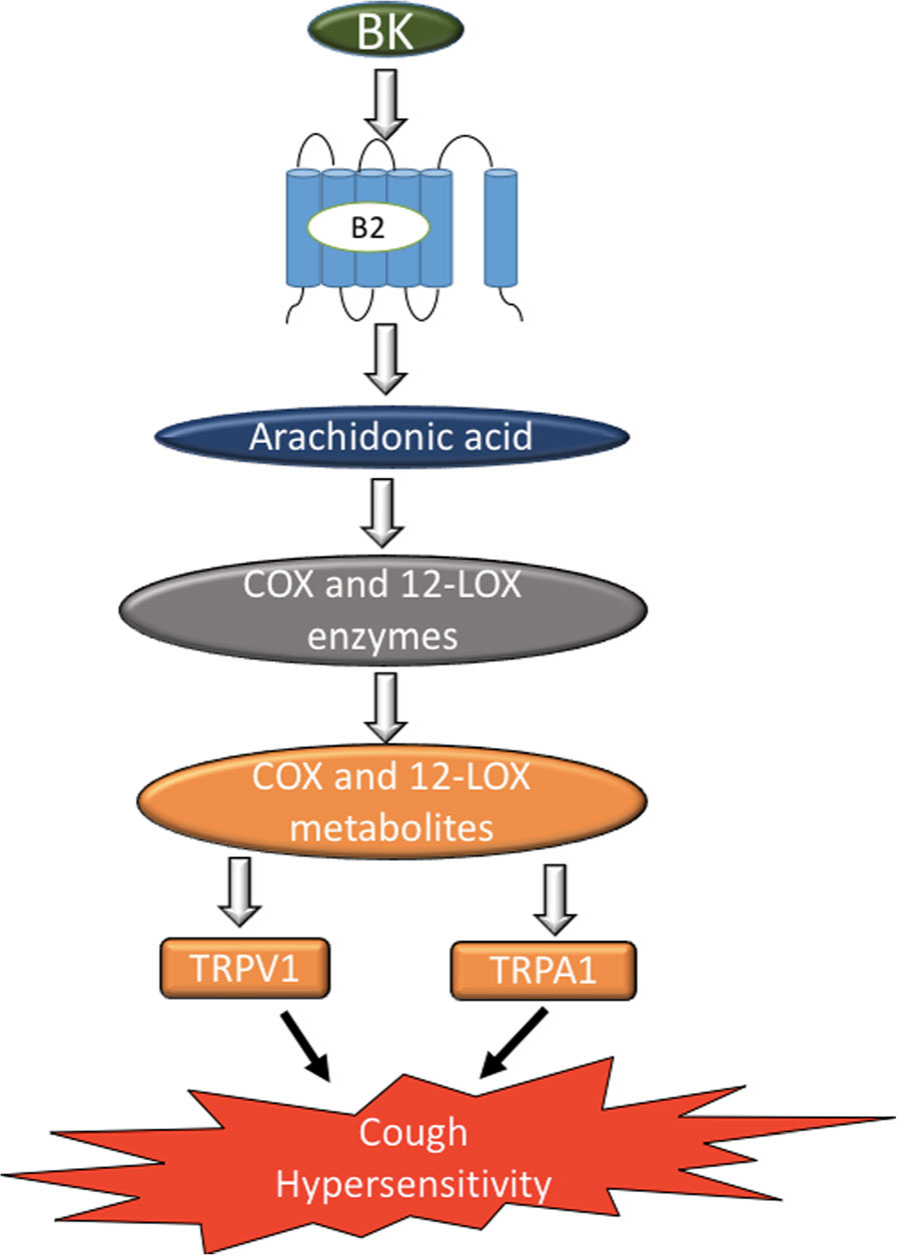
Summary of our findings and a proposed mechanism by which BK sensitize the cough reflex centrally. Bradykinin acts on B_2_ receptors (B_2_R) on second order neurons to stimulate the release of COX and 12-LOX metabolites which in turn activate TRPV1 and TRPA1 channels on the second order neurons resulting in an enhanced cough response

In summary, our data show that centrally administered BK activates B_2_ receptors which results in enhanced citric acid-induced cough and airway obstruction via the sensitization of TRPV1 and/or TRPA1 channels through metabolites of COX and/or 12-LOX (Fig. 10). Collectively, our findings point to an important role for BK, via B_2_ receptors, in the central sensitization of airway responses namely cough and airway obstruction and further identify TRPV1, TRPA1 and metabolites of COX and 12-LOX as key molecules in the sensitization process. An important finding in this study is that simultaneous blockade of TRPV1 and TRPA1 results in a synergistic inhibitory effect on cough and airway obstruction.

## Conclusion

The findings in this study may have relevance to patients with chronic cough where their demonstrated CHS could be, at least, partly due to enhanced central activity of the BK/B_2 _receptor/TRPV1/TRPA1 signaling pathway and suggest that blockade of central B_2_ receptors or concurrent inhibition of TRPV1/TRPA1 channels or COX/12-LOX enzymes may offer novel therapeutic treatment approaches for these patients.

## Abbreviations

ACSF: Artificial cerebrospinal fluid
AMP: Adenosine 5′-monophosphate
AMPA: α-amino-3-hydroxy-5-methyl-4-isoxazolepropionic acid
ANOVA: One Way Analysis of Variance
AP: Anteroposterior
AUC: Area under the curve
AVPNs: Airway-related vagal preganglionic neurons
BK: Bradykinin
CHS: Cough hypersensitivity syndrome
CNS: Central nervous system
DRG: Dorsal root ganglia
DV: Dorsoventral
EPSCs: Excitatory postsynaptic currents
i.c.v.: intracerebroventricularly
ML: Mediolateral
NGF: Nerve growth factor
NIH: National Health Institute
nTS: nucleus tractus solitarius
PGD_2_: Prostaglandin D_2_
PGE_2_: Prostaglandin E_2_
SP: Subtance P
